# Mutations or copy number losses of *CD58* and *TP53* genes in diffuse large B cell lymphoma are independent unfavorable prognostic factors

**DOI:** 10.18632/oncotarget.13065

**Published:** 2016-11-04

**Authors:** Yang Cao, Tao Zhu, Peiling Zhang, Min Xiao, Shuhua Yi, Yan Yang, Qinlu Li, Shaoping Ling, Yafei Wang, Lili Gao, Li Zhu, Jue Wang, Na Wang, Liang Huang, Peihong Zhang, Qiongli Zhai, Lugui Qiu, Jianfeng Zhou

**Affiliations:** ^1^ Department of Hematology & Cancer Biology Research Center, Tongji Hospital, Tongji Medical College, Huazhong University of Science and Technology, Wuhan, Hubei, P.R. China; ^2^ State Key Laboratory of Experimental Hematology, Institute of Hematology and Blood Diseases Hospital, Chinese Academy of Medical Sciences and Peking Union Medical College, Tianjin, P.R. China; ^3^ Laboratory of Genome Variations and Precision Bio-Medicine, Beijing Institute of Genomics, Chinese Academy of Sciences, Beijing, P.R. China; ^4^ Tianjin Cancer Hospital, Tianjin Medical University, Tianjin, P.R. China

**Keywords:** TP53, CD58, mutation, copy number loss, DLBCL

## Abstract

The advent of next generation sequencing (NGS) technologies has expedited the discovery of novel genetic lesions in DLBCL. The prognostic significance of these identified gene mutations is largely unknown. In this study, we performed NGS for the 27 genes most frequently implicated in 196 patients. Interestingly, *TP53* mutations were found to be significantly more common in DLBCL with *MYC* translocations (*r* = 0.446, *P* = 0.034). While no gene mutation was found to be more prevalent in patients with DLBCL with bone marrow involvement, *MYD88* mutations were more common in primary DLBCL of the CNS or testis. To evaluate the prognostic significance of the abnormalities of these 27 genes, a total of 165 patients with newly diagnosed DLBCL, NOS were included in a multivariate survival analysis. Surprisingly, in addition to the *TP53* mutation, *CD58* mutation was found to predict poor clinical outcome. Furthermore, copy number loss of *CD58* or *TP53* was also identified to be an independent negative prognostic factor. Our results have uncovered the previously unknown critical impact of gene mutations on the prognosis of DLBCL and are fundamentally important for the future design of tailored therapy for improved clinical outcomes.

## INTRODUCTION

Diffuse large B-cell lymphoma (DLBCL) is the most common subtype of non-Hodgkin lymphoma (NHL), accounting for more than 30% of all newly diagnosed NHL [1]. Although DLBCL is generally readily curable with the current first line chemotherapy regimen, rituximab plus cyclophosphamide, doxorubicin, vincristine, and prednisone (R-CHOP), approximately 40% of the patients will eventually die of relapse or refractory disease even after rescue with high-dose chemotherapy and/or autologous hematopoietic stem cell transplantation. The fact that DLBCL has been classified into more than 15 subgroups in the 2008 version of the World Health Organization (WHO) classification of lymphoid malignancies underscores the heterogeneity of DLBCL with respect to their distinct morphologic, biologic, immunophenotypic, and clinical characteristics.

To date, predictors for the risk stratification of DLBCL include clinical prognostic factors, staging and evaluation of the response to therapy by positron emission tomographic scans (PET-CT), gene expression profiling (GEP) and molecular prognostic factors [2–5]. The International Prognostic Index (IPI) has long been the primary clinical tool to predict the prognosis of patients with aggressive NHL. Whereas IPI is easily applied for stratification purpose, it does not provide the necessary biologic insight needed for individualized therapy approaches containing novel targeted agents. GEP has helped to stratify DLBCL into the biologically and prognostically relevant subgroups of relevant two main subgroups of the germinal center B-cell-like (GCB) and the activated B cell-like (ABC) DLBCL. Patients with the GCB subtype have a superior overall survival compared with the ABC subtype with R-CHOP-based treatment [4]. Importantly, the GEP stratification has revealed that the defined subgroups are associated with dysfunction in distinct oncogenic pathways that can be specifically targeted for an improved clinical outcome. Furthermore, some molecular cytogenetic aberrations such as *MYC* translocation and/or amplification are poor prognostic factors, independent of the IPI status [6–9]. The clinical success of dose-adjusted etoposide, prednisone, vincristine, cyclophosphamide, and doxorubicin (DA-EPOCH) over R-CHOP in treating patients with *MYC*+ DLBCL has highlighted the profound impact of molecular aberrations on clinical management for an improved clinical outcome [10].

For the past several years, the advances in high-throughput sequencing technologies have made it possible to characterize DLBCL at the whole genome level rather than by using a candidate gene approach. The application of targeted next generation sequencing (NGS) to DLBCL has already yielded many important discoveries in an unbiased and comprehensive manner. The identification of structural alterations and corresponding deregulated signaling pathways has unveiled an unprecedented understanding of DLBCL pathogenesis that will provide invaluable preclinical rationale for a more precise treatment of DLBCL. On the other hand, the biological and prognostic significance of these gene mutations on DLBCL is largely unknown. Previously, several studies have convincingly shown that the *TP53* mutation is an independent negative prognostic biomarker in DLBCL [11]. With detailed landscapes of DLBCL genomes are emerging, there is an urgent need to define the clinical significance of these identified individual genetic abnormalities.

In this study, we performed NGS for the 27 genes most frequently implicated in 196 patients with newly diagnosed DLBCL. A total of 165 patients with newly diagnosed DLBCL, NOS were included in a multivariate analysis to identify the gene mutations having independent prognostic value. Our results have uncovered the previously unknown critical impact of some gene mutations on the prognosis of DLBCL and are fundamentally important for the future design of tailored therapy for improved clinical outcome.

## RESULTS

### Patient cohort and characteristics

A total 196 patients with DLBCL and 6 reactive lymph node samples were successfully analyzed by NGS for the 27 genes most frequently implicated in DLBCL in previous studies [12, 13]. Complete follow-up information including response to initial therapy, overall survival (OS) and progression-free survival (PFS), were available in 177 of the 196 patients. Of these 177 DLBCL patients, 165 cases were diagnosed as DLBCL, NOS and the other 12 cases were diagnosed with primary DLBCL of the CNS or testis. The clinical characteristics of 177 DLBCL patients are summarized in Table 1. A total of 165 patients with newly diagnosed DLBCL who had been treated with R-CHOP-like regimens following the NCCN guideline were finally included in the study for identification of the gene mutations with independent prognostic significance in DLBCL, NOS. The median observation period (from the day of diagnosis to the final observation date) for the entire study population was 24.0 months. Of all 165 DLBCL patients, the median age at diagnosis was 54 years old (range, 14 to 83), and 29.10 % of the patients were over 60 years old. Eighty nine DLBCL cases (53.94%) were males and 76 (46.06 %) were females. B symptoms were observed in 49 patients (33.1 %). Seventy eight cases (47.27 %) were Ann Arbor stage I-II and 87 cases (52.73 %) were stage III-IV. Elevated serum LDH levels were observed in 82 cases (46.63%). Forty seven patients (28.48 %) were found to have ≥2 extranodal sites involvement, and 10 cases patients (6.06 %) presented with massive mass at diagnosis. Other risk factors, including ECOG and response to therapy, are summarized in Table 1.

**Table 1:** Clinical characteristics of 177 patients with DLBCL

Clinical characteristic		DLBCL (NOS)		Primary DLBCL of CNS or Testis	
		Cases	Percent (%)	Cases	Percent (%)
Age	<60	117	70.90	10	83.33
	>=60	48	29.10	2	16.67
Gender	Female	76	46.06	5	41.67
	Male	89	53.94	7	58.33
Ann Arbor stage	1~2	78	47.27	4	33.33
	3~4	87	52.73	8	66.67
B symptoms	No	101	61.21	9	75.00
	Yes	64	38.79	3	25.00
LDH	Normal	74	44.85	9	75.00
	Elevated	91	55.15	3	25.00
ECOG	0~1	123	74.55	8	66.67
	>=2	42	25.45	4	33.33
Massive mass	No	155	93.94	12	100.00
	Yes	10	6.06	0	0.00
IPI	0~1	74	44.85	6	50.00
	2~3	73	44.24	5	41.67
	4~5	18	10.91	1	8.33
Extranodal involvement	0-1	118	71.52	8	66.67
	>=2	47	28.48	4	33.33
Response*	CR	77	46.67	5	41.67
	PR	61	36.97	4	33.33
	SD	6	3.63	0	0.00
	PD	21	12.73	3	25.00

* assessed after 4 cycles of chemotherapy

### Next generation sequencing data

For NGS, each individual sample library was established and sequenced on an eight-lane PicoTiterPlate (Roche) after confirmation of its quality. Supplementary Figure S1A illustrates a representative distribution of amplicon reads for one DLBCL sample. On average, approximately 5, 880, 840 sequencing beads (key pass wells) per patient were generated, which yielded a median of 299,744 high-quality sequencing reads (passed filter wells). The median length of reads per individual sample ranged between 75 and 150 base pairs (bp). A median of 29.2 mega bp was sequenced per patient. Supplementary Figure S1B demonstrates the overall distribution of amplicon coverage for the 203 samples. Box plots are shown for each of the corresponding amplicons representing the 27 genes most frequently implicated in DLBCL. The median number of generated reads per amplicon was 3920-fold (range, 636- to 9,277-fold). Moreover, transitions accounted for approximately 60% of these events (Supplementary Figure S1C), similar to patterns observed in DLBCL [13]. Thus, the generated NGS data allowed a sensitive and reliable detection of variations. Furthermore, to validate the results of NGS, we subjected a total of 394 variants affecting 27 genes called by NGS to PCR-based Sanger sequencing. Of these 394 variants, 376 had been confirmed by Sanger sequencing, demonstrating a high concordance rate of 95.4% between NGS and Sanger sequencing. The discordant cases all occurred because NGS called a variant that was not supported by Sanger sequencing. We did not observe any cases where a variant determined to be absent by NGS was found to be actually present by Sanger sequencing (false negative). Therefore, we are highly confident that NGS calls for somatic variants are real because our NGS calls for absence of variants are highly accurate.

### Frequency and distribution of molecular mutations

**Figure 1:**
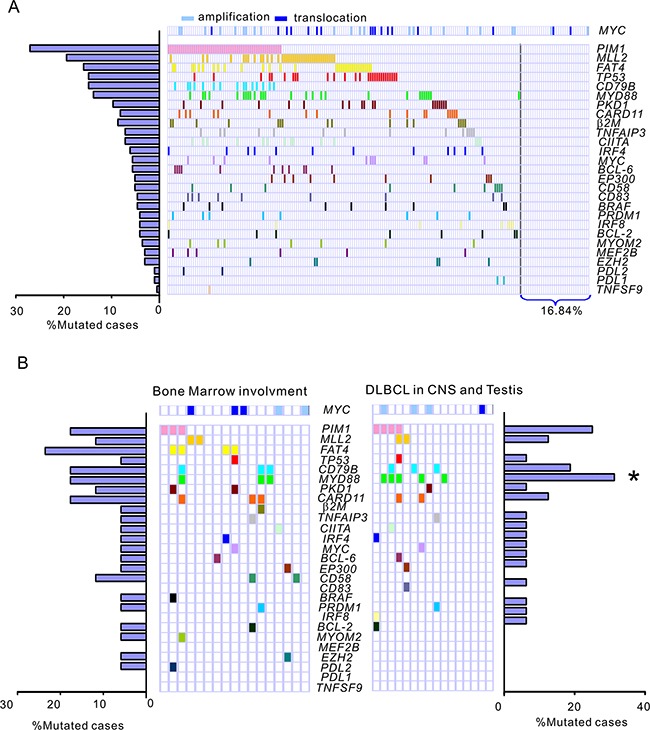
Frequencies and distribution of gene mutations in patients with DLBCL **A.** A total of 196 DLBCL cases were subjected to targeted next generation sequencing for the 27 genes most frequently implicated in DLBCL. The bar graph shows the proportion of a given gene mutation in the 196 DLBCL samples. The distribution of mutations is denoted in the cohort of patients with DLBCL. Each patient is represented by a box. A colored box indicates a case harboring at least 1 mutation in the given gene. The patient cohort is further annotated according to amplification (n = 23) and translocation (n = 14) of the *MYC* gene as determined by fluorescence *in situ* hybridization analysis on paraffin-embedded tissue sections. **B.** Prevalence and distribution of gene mutations in special DLBCL subgroups. Bone marrow involvement, DLBCL with confirmed infiltration of lymphoma cells involving the bone marrow. DLBCL in CNS and testis, primary DLBCL of the CNS or testis. *, *P* < 0.05, Primary DLBCL of the CNS or testis *versus* DLBCL, NOS.

Among the 196 DLBCL patients, mutations of the *PIM1* gene were detected in 53 cases (27.04 %),*MLL2* in 38 (18.39 %), *FAT4* in 34 (15.9 %), *TP53* in 29 (14.80 %), *CD79B* in 29 (14.80 %), *MYD88* in 27 (13.78 %), *PKD1* in 19 (9.69 %), *CARD11* in 17 (8.67%), *β_2_M* in 16 (8.16 %), *TNFAIP3* in 14 (7.14 %), *CIITA* in 14 (7.14 %), *IRF4* in 12 (6.12 %), *MYC* in 11 (5.61 %), *BCL-6* in 11 (5.61 %), *CD58* in 10 (5.10%) and *EP300* in 10 (5.10%). The remaining 12 genes, including *CD83*, *BRAF*, *PRDM1*, *IRF8*, *BCL-2*, *MYOM2*, *MEF2B*, *EZH2*, *PDL2*, *PDL1* and *TNFSF9*, had a mutation rate less than 5% (Figure 1A, Supplementary Table S1). Overall, 163 patients with DLBCL (83.16%) were found to harbor at least one gene mutation examined. Using FISH analysis on FFPE sections, *MYC* amplification and translocation were detected in 18.88% of patients (37/196), of which 11.73 % were *MYC* amplification (23/196) and 7.14% *MYC* translocation (14/196). Among the 14 patients with *MYC* translocation, 3 cases were shown to concurrently harbor *BCL-2* translocation (double hit). When the patient cohort was annotated according to *MYC* gene amplification and translocation, *TP53* mutations were found to significantly correlate with *MYC* translocations (*r* = 0.446, *P* = 0.035) but not amplification. In DLBCL with *MYC* translocation, 42.86% patients (6/14) harbored a *TP53* mutation, of whom, 2 cases had *BCL-2* translocation. In contrast, in DLBCL without *MYC* translocation, only 11.5% cases (21/182) were detected to harbor *TP53* mutation.

In DLBCL with bone marrow involvement, the most common mutations were *FAT4* (23.5%), *PIM1* (17.6%), *CARD11* (17.6%) and *MYD88* (17.6%). However, no prevalent gene mutation was detected in this subgroup compared to all the other DLBCL, NOS cases. In our series of 16 patients with primary DLBCL of the CNS or testis, *FAT4* mutation exhibited a lower frequency in primary DLBCL of the CNS or testis compared with all the other DLBCL patients analyzed (*P* = 0.055). Somatic mutations in *PIM1* (4/16, 25.0%), *CD79B* (3/16, 18.75%) and *MYD88* (5/16, 31.25%) were the most prevalent aberrations. Notably, all 5 *MYD88* mutation cases are L265P, which is consistent with the observation of Yamada *et al* [14]. Mutations of *MYD88* were significantly more frequent in primary DLBCL of the CNS or testis than that of all the other DLBCL, NOS cases (21/180, 11.7 %) (*P* = 0.034). In addition to 5 *MYD88* mutation cases, 1 more case was detected to have deletion of *MYD88* and no copy number gain was found in patients with primary DLBCL of the CNS or testis. To observe the correlation of *MYD88* mutations with the activation of associated BCR signaling pathway, the surrogate nuclear factor-κB (NF-κB) and P38-MAPK antigens in primary biopsies were analyzed by immunohistochemical (IHC) staining. The cases with *MYD88* L265P showed significantly elevated NF-κB activity compared with wild-type cases (for NF-κB1, *P* = 0.002, for NF-κB2, *P* = 0.048). The *MYD88* mutation cases also exhibited increased P38-MAPK activity in contrast to *MYD88* wild-type cases (*P* = 0.037)(Supplementary Figure S2).

### Mutations in *CD58* and *TP53* are associated with poor prognosis

**Figure 2:**
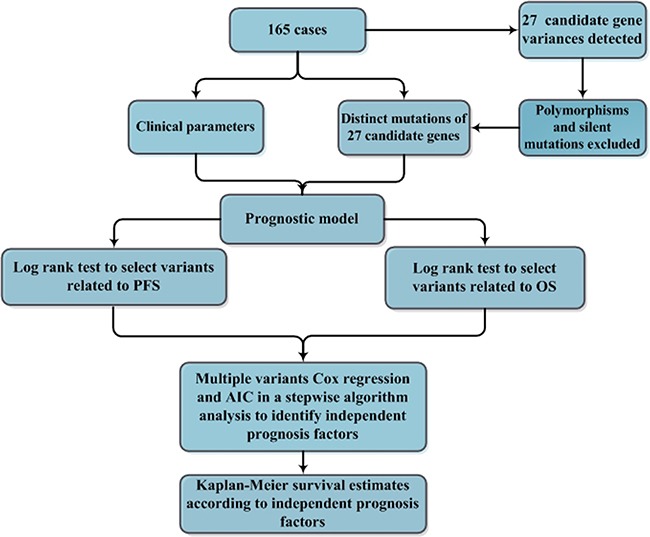
A flowchart for the identification of independent prognostic factors in patients with DLBCL A total of 165 cases of DLBCL, NOS were included in the analysis for identifying independent prognostic factors in patients with DLBCL. Polymorphisms and silent mutations of 27 gene variations detected by next generation sequencing in the DLBCL cases were excluded, and distinct mutations were confirmed by Sanger sequencing. To indentify the prognostic factors, the mutation status of the 27 genes and clinical features, including age, gender, B symptoms, massive mass, Ann Arbor stage, extranodal involvement, bone marrow involvement, ECOG, elevated LDH, *MYC* aberration (including translocation and amplification) were selected as candidate parameters. The selected parameters were initially analyzed by the *log*-rank test to identify potential univariate prognostic factors related to PFS or OS. The resulting parameters were further analyzed in a multivariate modeling by multiple variable Cox regression and the generalized Akaike Information Criterion (AIC) to identify the independent prognostic factors. The independent prognostic factors were finally analyzed by *log*-rank tests to distinguish the significant difference of their roles in PFS or OS estimated by the Kaplan-Meier method.

**Figure 3:**
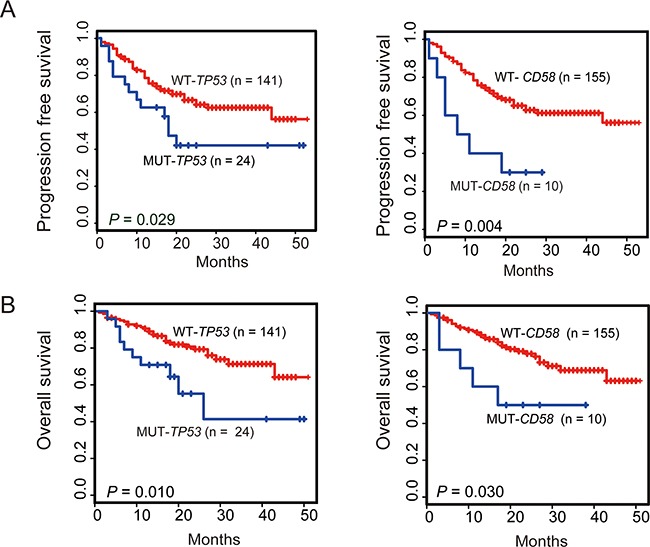
Kaplan-Meier survival estimates according to the mutation status of *CD58* or *TP53* Data are shown for PFS **A.** and OS **B.** of patients with DLBCL, NOS. Patients harboring gene mutations are indicated by blue lines. The red lines indicate patients with wild-type gene. Tick marks represent patients whose data were censored last time they were known to be progression-free or alive.

We next assessed the prognostic significance of gene mutations as well as the clinical features in patients with DLBCL with regard to overall survival (OS) and progression-free survival (PFS) (Figure 2). The mutational status of each of the 27 genes and the clinical features, including age, gender, B symptoms, massive mass, Ann Arbor stage, extranodal involvement, bone marrow involvement, ECOG performance status, elevated LDH, and *MYC* cytogenetic aberration (including translocation and amplification), cell of origin (COO) determined by IHC assessment were selected as candidate parameters. The selected parameters were analyzed by the *log*-rank test to identify potential prognostic variables related to PFS or OS. The result of the univariate analysis is summarized in Table 2. B symptoms (*P* = 0.019, *P* = 0.04, respectively) and bone marrow involvement (*P* = 0.036, *P* = 0.012, respectively) were significantly associated with both shorter PFS and OS. Patients with non-GCB subtype had significantly worse PFS (*P* = 0.033) and OS (*P* = 0.024) than those with GCB subtype. Ann Arbor stage and *MYC* aberration were significantly associated with poor PFS (*P* = 0.026, *P* = 0.016, respectively) and a non-significant trend towards shorter OS (*P* = 0.05, *P* = 0.158, respectively). Elevated LDH was associated with a non-significant tendency towards poor PFS (*P* = 0.112) and OS (*P* = 0.059). Interestingly, either of *CD58* or *TP53* gene mutation was a significant poor prognostic factor for both PFS (*P* = 0.004, *P* = 0.03, respectively) and OS (*P* = 0.03, *P* = 0.011, respectively) (Figure 3A–3B). In agreement with previous reports, *MYC* aberration was a significantly poor prognostic factor for both PFS and OS [6–9, 15]. We therefore selected individual clinical features (COO, Ann Arbor stage, B symptoms, bone marrow involvement, and *MYC* aberration) and mutation parameters (*CD58*, *TP53*) for further analysis in a multivariate model. Notably, Ann Arbor stage, bone marrow involvement or *MYC* cytogenetic aberration was no longer a significant poor independent prognostic factor in the Cox regression model. However, COO as well as B symptoms were found to be an independent factor predicting shorter PFS and OS. Multivariate analysis revealed that poor PFS and OS were present in patients with the *TP53* mutation (*P* = 0.004, *P* < 0.001, respectively). Interestingly, *CD58* mutation was significantly associated with a high risk of progression (HR = 3.75; 95% CI, 1.63-8.63; *P* = 0.002) and shorter OS (HR = 3.97; 95% CI, 1.49-10.57; *P* = 0.006) (Table 2).

**Table 2:** Analysis of the risk factors for clinical outcome

Univariate analysis	PFS	OS	Multivariate analysis	PFS		OS	
	*P*	*P*		HR (95% CI)	*P*	HR (95% CI)	*P*
*CD58* mutation	0.004	0.03	COO (non –GCB)	2.26 (1.22-4.18)	0.009	3.15 (1.43-6.91)	0.004
*TP53* mutation	0.03	0.011	B symptoms	1.89 (1.07-3.35)	0.029	2.36 (1.21-4.58)	0.011
Ann Arbor stage III/IV	0.026	0.05	*CD58* mutation	3.75 (1.63-8.63)	0.002	3.97 (1.49-10.57)	0.006
COO (non-GCB)	0.033	0.024	*TP53* mutation	2.85 (1.40-5.82)	0.004	4.84 (2.09-11.21)	< 0.001
B symptoms	0.019	0.04					
*MYC* aberration	0.016	0.158					
BM involvement	0.036	0.012					

Abbreviations: CI, confidence interval; HR, hazard ratio; OS, overall survival; PFS, progression-free survival

### Mutational pattern of *CD58* and *TP53* genes in DLBCL

**Figure 4:**
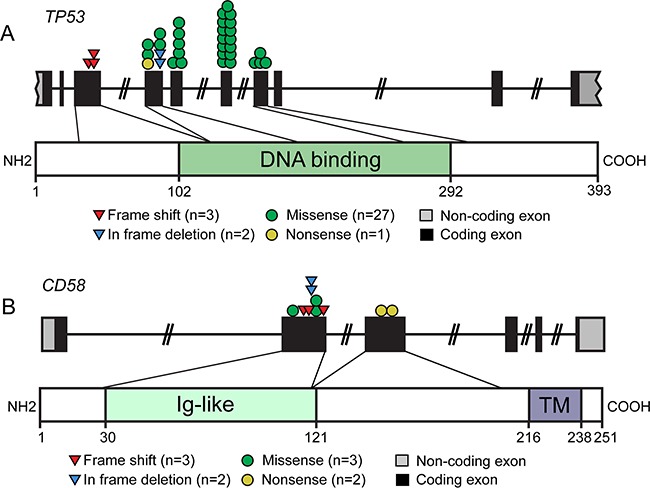
Schematic overview of the mutational pattern of *CD58* and *TP53* genes in DLBCL Distinct gene mutations in 165 cases of DLBCL, NOS were described. **A.** Schematic representation of the distribution of frameshift, missense, in frame deletion and nonsense mutations affecting the *TP53* genomic region. Mutations affecting the *TP53* gene were frequently located in the DNA binding domain. **B.** The distribution of frameshift, missense, in frame deletion and nonsense mutations affecting the *CD58* genomic region. Ig, single-pass type I membrane immunoglobulin; TM, transmembrane.

We further characterized the mutational pattern of *CD58* and *TP53* genes in 196 patients with DLBCL. The *TP53* gene is a crucial tumor suppressor that acts by transcription-dependent activity (TA) and transcription-independent activity (TIA) in the nucleus and cytoplasm, respectively [16]. *TP53* dysfunction has been implicated in lymphomagenesis and disease progression, and either of the normal function of TA and TIA of p53 is crucial for tumor suppression. The DNA-binding domain (DBD) of p53 is the most important domain for its TA and TIA2 [11]. All mutations identified in our study were referenced in the COSMIC database (*e.g.*, COSM44656, COSM12005). We observed that exons 4-7 of *TP53* were most often mutated (Figure 4A). Overall, 90% of *TP53* mutations occurred in the DBD. Investigation of the type of *TP53* mutation showed that the most common mutations were missense, followed by frameshift, in-frame and nonsense mutations. Functional prediction of the 27 missense mutations was analyzed by SIFT (http://sift.jcvi.org/) methods based on protein sequence homology. Over 90% missense mutations were predicted to be deleterious (i.e. disrupt the p53 function). Nonsense mutation (n = 1) and frameshift-inducing insertions and deletions (n = 3) led to the production of truncated proteins, indicating a deleterious effect. To examine the functional consequences of *TP53* mutations, we analyzed p53 expression in DLBCL biopsy. IHC for p53 revealed that p53 overexpression to be associated with mutational status (P < 0.001). Most tumors with *TP53* mutation were shown to express the protein at an elevated level (n = 12/17, 71.0%), whereas we detected an elevated p53 expression in fewer tumors with wild-type *TP53* (n = 3/23) (Supplementary Figure S3A). Survival analysis showed that p53 overexpression predicted worse PFS and OS (*P* = 0.001, *P* = 0.007, respectively) (Supplementary Figure S3B).

CD58 is known to be a member of the immunoglobulin superfamily that functions as a ligand of CD2 on T cells and natural killer lymphocytes (NK), participating in their adhesion and activation [17]. In our data, all mutations of the *CD58* gene occurred in DLBCL, NOS. Half of the mutations (n = 5/10) were nonsense mutations (n = 2) and frameshift insertion/deletions (n = 3) that resulted in aberrant transcripts encoding for truncated proteins, all of which lacked the CD58 transmembrane domain (Figure 4B). The missense mutations (n = 3) were predicted to be damaging by SIFT and Polyphen-2 (http://genetics.bwh.harvard.edu/pph2/) methods. Furthermore, the effect of the in-frame deletions (n = 2) on CD58 protein were predicted to be deleterious using PROVEAN software (http://provean.jcvi.org/seq_submit.php). To confirm the impact of these mutations on CD58 protein, IHC analysis of DLBCL biopsy was performed. Among the cases with *CD58* mutation, 80% (n = 2/10) exhibited negative protein expression, while only one wild type sample showed loss of CD58 surface protein expression (n = 15) (Supplementary Figure S4A). Patients lack CD58 protein expression had significantly shorter PFS and OS than those with CD58 expression (*P* = 0.045, *P* = 0.05 respectively) (Supplementary Figure S4B). These results strongly suggest a tumor-suppressive role of CD58 in DLBCL.

### Copy number loss of *CD58* or *TP53* predicts poor prognosis

**Figure 5:**
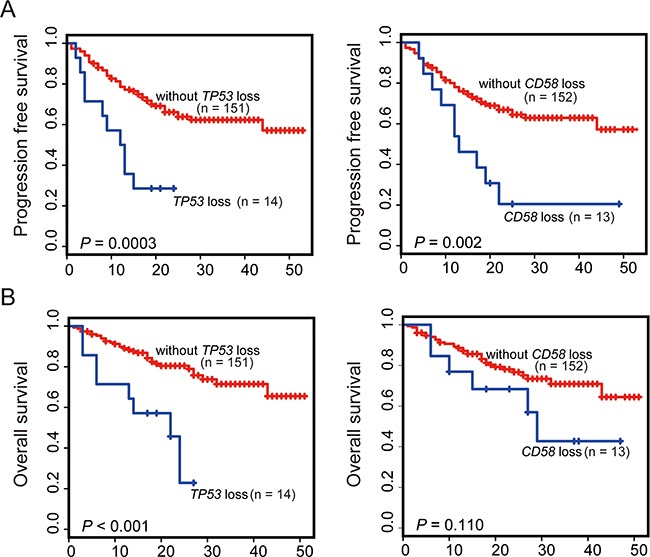
Kaplan-Meier survival estimates according to the copy number loss of *CD58* or *TP53* Data are shown for PFS **A.** and OS **B.** of patients with DLBCL (n = 165). Patients harboring gene copy number loss are indicated by blue lines. The red lines indicate patients without gene copy number losses. Tick marks represent patients whose data were censored last time they were known to be progression-free or alive.

In addition to gene mutation, copy number variation (CNV) is another common type of genetic variation. Given the important involvement of *CD58* and *TP53* gene mutation in the poor prognosis of DLBCL, we investigated the association of *CD58* and *TP53* copy number variants with the prognosis of DLBCL. Copy number variations of *CD58* and *TP53* in 196 DLBCL samples were analyzed based on read-depth information and heterozygous sites. A normalized distribution of the mean depth ratio with standard deviation for the *CD58* or *TP53* gene was generated based on NGS data derived from 203 samples (including 6 reactive lymph node samples and 1 duplicate DLBCL sample). To determine whether a sample had a loss or gain of copy number for a given gene, the depth ratio of the sample was compared with the normalized mean depth ratio of all 203 samples. As shown in Supplementary Figure S2, by using this NGS data-based analysis, CNV, including copy number gain or loss of the *CD58* or *TP53* genes, was successfully identified. The results showed that copy number loss of *TP53* was detected in the majority of cases (16/18), whereas copy number gain of *TP53* was found only in 2 out of 18 cases. Considering that both gene mutation and copy number loss of *TP53* as well as *CD58* would lead to loss of function of these genes, we define both types of gene aberrations as a *TP53* or *CD58* abnormality. Among patients with *TP53* mutations (n = 29) or copy number loss (n = 16), 2 cases were found to simultaneously harbor a *TP53* mutation and loss of copy number. Therefore, the total frequency of *TP53* abnormalities was detected in 21.94% of cases of DLBCL. When the patient cohort was further annotated according to *MYC* gene amplification and translocation, no significant correlation was found between copy number loss of *TP53* and *MYC* translocation or amplification (*P* = 0.39, *P* = 0.10, respectively). Copy number loss of *CD58* occurred in the majority of cases (14/21), whereas gains occurred in fewer cases (7/21). Interestingly, almost all instances of copy number loss and *CD58* gene mutations occurred in patients with DLBCL, NOS (13/14, respectively). Among patients with *CD58* mutation (n = 10) or copy number loss (n = 14), two cases were found to simultaneously harbor *CD58* mutation and loss of copy number. Therefore, the total frequency of CD58 abnormalities was detected in 12.22% of patients with DLBCL, NOS. To address whether copy number loss of *CD58* or *TP53* was prognostic, copy number loss of the *CD58* or *TP53* gene was entered into previous prognostic models as a new potential parameter for further analysis. In univariate analysis (Figure 5), copy number loss of *TP53* gene was a poor prognostic factor for both PFS and OS. Patients with copy number loss of *TP53* had significantly shorter overall survival times (*P* < 0.001) than those without copy number loss. CNV of *CD58* was significantly associated with poor PFS (*P* = 0.002) and a non-significant trend toward shorter overall survival compared with patients without copy number loss of *CD58* (*P* = 0.11). Therefore, copy number loss of *CD58* or *TP53* was chosen in multivariate model analysis. When adjusted in multivariate Cox regression analysis, independent prognostic factors included COO, B symptoms, *CD58* or *TP53* mutations and copy number loss of *CD58* or *TP53*. The results of the initial analysis of independent prognostic factors associated with either PFS or OS are shown in Table 3. Copy number loss of *TP53* was found to be an independent unfavorable prognostic factor for both PFS (HR = 2.54; 95% CI, 1.19-5.42; *P* = 0.016) and OS (HR = 3.87; 95% CI, 1.70-8.81; *P* = 0.010). Patients who had copy number loss of CD58 were found to have an independent adverse impact on PFS (HR = 2.61; 95% CI, 1.22- 5.56; *P* = 0.013).

**Table 3:** Analysis of the risk factors for clinical outcome after inclusion of copy number losses as new parameters

Univariate analysis	PFS	OS	Multivariate analysis	PFS		OS	
	*P*	*P*		HR (95% CI)	*P*	HR (95% CI)	*P*
*CD58* mutation	0.004	0.03	COO (non-GCB)	1.86 (1.00-3.45)	0.048	2.80 (1.29-6.09)	0.009
*TP53* mutation	0.03	0.011	B symptoms	2.07 (1.17-3.67)	0.012	1.98 (1.03-3.79)	0.04
Ann Arbor stage III/IV	0.026	0.05	*CD58* mutation	3.85 (1.61-9.18)	0.002	4.72 (1.74-12.80)	0.002
COO (non-GCB)	0.033	0.024	*TP53* mutation	3.07 (1.52-6.19)	0.002	4.94 (2.15-11.32)	< 0.001
B symptoms	0.019	0.04	CNVs of *CD58*	2.61 (1.22-5.56)	0.013	NS	NS
*MYC* aberration	0.016	0.158	CNVs of *TP53*	2.54 (1.19-5.42)	0.016	3.87 (1.70-8.81)	0.01
BM involvement	0.036	0.012					
CNVs of *CD58*	0.002	0.11					
CNVs of *TP53*	< 0.001	< 0.001					

Abbreviations: CI, confidence interval; HR, hazard ratio; OS, overall survival; PFS, progression-free survival

## DISCUSSION

In this study, we performed NGS for 27 genes most frequently implicated in 196 cases of newly diagnosed DLBCL followed by validation using Sanger sequencing. In general, the mutated frequencies of the examined genes were comparable to those reported previously. The most frequently mutated genes identified in our cohort were *PIM1*, *MLL2*, *FAT4*, *TP53* and *CD79B*, which is consistent with previous reports [12, 13, 18]. On the other hand, a few genes, including *MYOM2*, *PRDM1* and *MEF2B*, exhibited relatively low mutational incidences in this study compared with those reported by Morin *et al* [18]. This observation supports the necessity to further characterize the mutational profiles of DLBCL among different populations.

Previously, *MYC* cytogenetic aberrations had been reported in 10–15% of DLBCL patients, which was generally taken as an independent indicator of poor prognosis [6–10]. Nevertheless, the prognostic value of isolated *MYC* translocation (single-hit lymphoma, SHL) has remained controversial due to the limited studies with conflicting conclusions [19, 20]. In this study, 18.87% of patients were found to have *MYC* cytogenetic aberrations, of which 11.73 % was *MYC* amplification and 7.14% *MYC* translocation. Interestingly, DLBCL with *MYC* translocation was found to have a significantly higher frequency of *TP53* mutation. Numerous studies have demonstrated that *MYC* abnormalities alone are not sufficient for lymphomagenesis [21, 22]. Other genetic alterations must be present in order to overcome the proapoptotic effects of *MYC.* The presence of *TP53* mutation would likely serve as the secondary event by promoting proliferation and survival of lymphoma cells. Notably, Gebauer, et al had recently performed NGS to investigate the mutational status of *TP53* in double hit lymphoma (DHL) and correlated genomic data with immunohistochemical reactivity for p53. They identified *TP53* mutations are frequent events in *MYC+/BCL-2+* lymphomas. Their findings are very consistent with our clinical data and strongly support our conclusion [23]. This previously unrecognized association of *MYC* translocation with *TP53* mutation argues that the presence of *MYC* cytogenetic aberrations per se may not be the most accurate prognostic factor. Other concurrent cytogenetic aberrations such as *BCL-2*,*BCL-6* translocation as well as *TP53* mutation should be simultaneously determined to provide more precise risk stratification when using *MYC* cytogenetic aberrations as biomarkers.

In the current study, mutational profiles were examined in special entities of DLBCL, including DLBCL with bone marrow involvement as well as DLBCL in immune-privileged sites (CNS and testis). In the present study, the BM infiltration was detected in 11.1% of patients with DLBCL, NOS (20/180). No significant difference in mutational frequencies of 27 genes was found between the DLBCL subgroups with or without BM involvement. In primary DLBCL of the CNS or testis, the cell cycle regulator *PIM1* and the regulators (*MYD88* and *CD79B*) of NF-κB pathway were the most frequently mutated genes, which are consistent with the recent data of Bruno *et* al [24]. The prevalence of *MYD88* mutation in primary DLBCL of the CNS or testis was significantly higher than previously reported studies in the whole DLBCL series ranging from 6.5% to 19.3% but similar to the observation in ABC-DLBCL (39%) [13, 18, 25–27]. *MYD88* has also been recurrently found mutated in majority of lymphoplasmacytic lymphoma (> 90%) [28, 29]. The L265P mutation was by far the most common variant observed in above lymphoid neoplasm. The association of *MYD88* L265P with survival in primary DLBCL of the CNS or testis remained unclear, although a worse outcome was raised in DLBCL, NOS. Furthermore, *MYD88* L265P mutation cases were found increased activity of associated BCR signaling pathway in primary DLBCL of the CNS or testis. The findings that mutation of *MYD88* was almost three times more common in primary DLBCL of the CNS or testis than that detected in DLBCL, NOS and the activation of associated BCR signal pathway in *MYD88* mutation cases suggested that MYD88 might be a potential target in primary DLBCL of the CNS or testis.

The most significant finding in the present study was the identification of *TP53* and *CD58* abnormalities as independent unfavorable prognostic factors based upon a stepwise regression multivariate analysis. The frequency of *TP53* mutation was in agreement with previous reports (10–23%), while the incidence of *TP53* deletion was lower than previously reported as determined by FISH [30]. *TP53* mutation in DLBCL has been consistently associated with a poorer clinical outcome among different studies, while the data regarding and the influence of *TP53* deletion on prognosis has remained controversial [30–32].

The conflicting results might be related to inter-observer variability and to differences in methodology and criteria in defining *TP53* deletion. In this study, copy number loss of *TP53* was defined using NGS-based analysis which takes the subclone loading into account. In a multivariate analysis, copy number loss of *TP53* was an independent unfavorable predictor of PFS and OS. Nevertheless, a standardized cut-off should be explored in future studies for routine clinical practice. In our patient cohort, the frequency of *CD58* mutation detected was in agreement with a previous report, while the rate of *CD58* deletion was lower than that reported elsewhere [12]. Notably, although gene mutations involved in immune recognition, such as *β_2_M*, *CIITA* and *CD83*, were common in our study, only *CD58* abnormality showed prognostic significance. Interestingly, *CD58* deletion predicted a poor PFS but only a non-significant trend toward a shorter overall survival (*P* = 0.11), which is possibly due to a short period of follow-up. These findings strongly support the critical roles of both the adaptive and innate immune evasion in lymphoma prognosis.

The accurate detection of somatic CNV is an essential part of cancer genome analysis. Current approaches to identify gene CNVs usually employ cytogenetic technologies, such as karyotyping, FISH and single-nucleotide polymorphism (SNP) array [33, 34]. On the other hand, NGS has evolved into a popular strategy for the comprehensive characterization of CNV over the last few years [35]. In fact, the resolution of NGS-based CNV detection has proven to be comparable with aCGH and SNP methods [36, 37]. DLBCL is usually identified with multiple subclones with heterogeneous genetic abnormalities. Therefore, NGS analysis could only identify CNVs where the proportion of malignant subclone of a given gene CNV is high enough [38]. The findings that *TP53* or *CD58* deletion was independent unfavorable predictor of clinical outcome in a multivariate analysis are encouraging enough to warrant a prospective study of these biomarkers.

## MATERIALS AND METHODS

### Sample selection and DNA extraction

Formalin-fixed paraffin-embedded (FFPE) diagnostic biopsies of 235 patients with DLBCL between July 2005 and December 2013 were retrospectively collected from TongJi Hospital and Institute of Hematology and Blood Diseases Hospital, Chinese Academy of Medical Sciences Medical Centers. These tissue samples had been obtained prior to any chemotherapy or radiotherapy. Patient samples were selected based on the following criteria: (1) diagnosis of DLBCL clinical stages I to IV; (2) having completed standard first-line chemotherapy recommended by NCCN; (3) at least 14 years of age at diagnosis; (4) availability of FFPE blocks of the diagnostic biopsies for DNA extraction. Cases were excluded if patients had a history of low grade B-cell lymphoma. The diagnosis of DLBCL for these patients was based on the morphological and immunohistochemical examination of biopsy material according to the WHO classification criteria and confirmed by two hematopathologists. Samples of 6 patients with reactive hyperplastic lymphadenitis were collected as negative controls. Appropriate informed consent was obtained from all donors prior to specimen collection in accordance with the Declaration of Helsinki and under a research protocol approved by the ethics committees of Tongji Hospital and Blood Diseases Hospital, Chinese Academy of Medical Sciences Medical Centers.

Genomic DNA was extracted from 3 to 5 μm thick FFPE sections of these patients with DLBCL and reactive hyperplastic lymphadenitis using the QIAamp DNA FFPE Tissue Kit (Qiagen) following the manufacturer's instructions. The tumor involvement in the FFPE sections was assessed to be at least 90% based on morphology and immunohistochemistry. The concentrations of DNA samples were measured using a NanoDrop spectrophotometer (Thermo Fisher Scientific) and the quality of DNA samples was examined by gel electrophoresis. Eleven samples of DLBCL were excluded due to poor DNA quality (n = 10) or misdiagnosis (n = 1).

### AmpliSeq-based mutation detection

Somatic mutations of selected samples from 224 patients with DLBCL and 6 reactive lymph nodes were identified by next-generation sequencing (NGS) using Ion AmpliSeq technology based on the Ion Torrent PGM platform (Life Technologies). For the detection of somatic mutations, a custom Ion AmpliSeq panel was designed using Ion AmpliSeq designer (www.ampliseq.com) to target 27 candidate genes, including *β_2_M, BCL2, BCL6, BRAF, CARD11, CD58, CD79B, CD83, CIITA, EP300, EZH2, FAT4, IRF4, IRF8, MEF2B, MLL2, MYC, MYD88, MYOM2, PDL1, PDL2, PIM1, PKD1, PRDM1, TNFAIP3, TNFSF9 and TP53* which are frequently implicated in previous studies [12, 13]. In the panel, two pools of 1184 amplicons encompassed the entire exome of these genes (padding ± 10 bp). The overall coverage rate was 95.1%. Based on the manufacturer's recommendations, 10 ng of genomic DNA was used for library preparation using Ion AmpliSeq Library kits 2.0 (Life Technologies). The libraries were quantified using the Invitrogen Qubit 2.0 Fluorometer (Life Technologies). Most of the samples yielded adequate libraries but failed in 28 cases of DLBCL. The libraries were then transferred to the Ion OneTouch 2 system to prepare templates using Ion PI Template OT2 200 Kit v2. Sequencing was carried out on the Ion Proton Sequencer using Ion PI Sequencing 200 Kit v2 (Life Technologies) according to the protocol. Quality control was performed using the Ion Sphere Quality Control Kit ensuring that 10–30% of template positive Ion Sphere particles (ISP) were targeted in the emPCR reaction. Torrent Suite Software 4.0.2 was used for data analysis. After alignment to Human Genome GRCh37 using Torrent Mapping Alignment Program 4.0.6, variant calling was performed using the Variant Caller plug-in. For variant detection, sequencing coverage of 100 × was needed, and genomic positions of low quality and variations with less than a 15% variant frequency were excluded. The selected variants were filtered, discarding somatic variants that correspond to known variants present in either dbSNP or the 1000 Genome Project. All variants were also subjected to prediction score programs from four algorithms (SIFT, Polyphen2, LTR and Mutation Taster) for every potential non-synonymous SNP in the human genome.

### Patients information

A total of 196 patients with DLBCL and 6 reactive lymph nodes were successfully analyzed by AmpliSeq-based mutation sequencing. The clinical, laboratory, and treatment data of these patients were gathered from medical records, including age, sex, ECOG performance status, the presence or absence of B symptoms, stage according to the Ann Arbor staging system, serum level of LDH, complete blood count, bone marrow biopsy and flow cytometry, extranodal sites involved, and radiographic examinations. Immunohistochemistry (IHC) based on staining of biomarkers including CD10, BCL-6 and MUM1 was performed to classify DLBCL patients into GCB and non-GCB subtype according to the Hans algorithm. To define bulky disease, we used a maximum diameter of the involved sites ≥ 10 cm. The performance status was assessed using ECOG criteria. The treatment response was assessed after 4 cycles of chemotherapy and classified as Complete Response (CR), Partial Response (PR), Stable Disease (SD) or Progressive Disease (PD) according to the International Working Group criteria. DLBCL cases suspected of BM involvement by lymphoma on BM smears were confirmed by bone marrow biopsy, immunohistochemistry and flow cytometry analysis. Primary DLBCL of the CNS (n = 9) or testis (n = 7) were diagnosed based on biopsy pathology and radiographic examination according to WHO classification criteria.

### Statistical analysis

The χ2 test, Fisher's exact test and Pearson's correlation were used to determine the correlation between subgroups of patients according to their gene mutational status. Overall survival (OS) was measured from the date of diagnosis to the date of death from any cause, or the latest follow-up. Progression-free survival (PFS) was determined from the date of diagnosis to the date of the first relapse or disease-related death or latest follow-up. To identify potential prognostic factors, the univariate comparison of OS and PFS was made. Differences were assessed using the *log*-rank test. All variables with a *P* value <0.05 in the univariate analysis were included in a stepwise regression multivariate analysis. The Cox model and the generalized Akaike Information Criterion (AIC) in a stepwise algorithm were used to determine hazard ratios (HRs), CIs and whether a variable was an independent prognostic factor for DLBCL. OS and PFS curves of different groups were analyzed using the Kaplan-Meier method. An association was considered statistically significant if *P*<0.05. All statistical analyses were carried out using free R software (ver. 3.1.1) with the survival, rms, MASS, DMwR, rpart packages along with some base packages.

## SUPPLEMENTARY MATERIALS AND METHODS


