# Supplementary material for: Mutations or copy number losses of CD58 and TP53 genes in diffuse large B cell lymphoma are independent unfavorable prognostic factors
Source: Oncotarget. 2016 Nov 4;7(50):83294–307. doi: 10.18632/oncotarget.13065 (PMC5347770; doi:10.18632/oncotarget.13065)
Supplement: Supplementary file 1 [file oncotarget-07-83294-s001.pdf]

Mutations or copy number losses of *CD58* and *TP53* genes in diffuse large B cell lymphoma are independent unfavorable prognostic factors

SUPPLEMENTARY MATERIALS AND METHODS

Mutation validation

A total of 394 variants of 27 genes in 196 DLBCL samples were detected by the next generation sequencing approach and confirmed by bidirectional Sanger sequencing. Genomic DNA was amplified by PCR using the high fidelity and efficient PCR enzyme KOD FX (TOYOBO) and sequenced using a 3500 Genetic Analyzer (Life Technologies) with a BigDye® Direct Cycle Sequencing Kit (Life Technologies). The analysis of chromatogram data was manually performed on Chromas (Technelysium Pty Ltd) and DNA Baser Sequence Assembler v4 (HeracleBioSoft). All primer sequences ordered from TsingKe, Wuhan, China are summarized as follows.

Primers names	Primers sequences
β_2M -E1-F	TCC CTC TCT CTA ACC TGG CAC
β_2M -E1-R	ACT TGG AGA AGG GAA GTC ACG
β_2M -E2-F	AGC TTG ACA CCA AGT TAG CCC
β_2M -E2-R	GAT GGG ATG GGA CTC ATT CAG
β_2M -E3-F	GAG GCT AGT AGG AAG GGC TTG
β_2M -E3-R	CAC TCC ACA GGA GAA GGG AAC
BCL2-E2-1-F	CCA AGA ATG CAA AGC ACA TCC
BCL2-E2-1-R	CCC AGC CTC CGTTAT CCT G
BCL2-E2-2-F	TGG AGG AGC TCT TCA GGG AC
BCL2-E2-2-R	TTG TCC AGA GGA GGA GGT AGG
BCL6-E4-F	CCG TAG AAG TTC TGG AGG TAG G
BCL6-E4-R	TGT GGG TTG CAG CAT ATC ATA G
BCL6-E5-F	ATT CCA TGT ACA GCC ACC TCC
BCL6-E5-R	GAG GAT GCA GGC ATT CTT ACT G
BCL6-E7-F	ACA GAG GAG AGC GGC CTT AG
BCL6-E7-R	CAC TAA GCA AAG ACA AGA CAG GG
BCL6-E8-F	CTT AGA AAT CCA GGA GCC ACG
BCL6-E8-R	GGA CTG AGT GGG ACT TTC TCC
BRAF-E10-F	TTT GGC CCA TCC TTT CCA
BRAF-E10-R	GCA GTG CCG TAG AAA TAT GCT T
BRAF-E14-F	CAT GGA TAA ATA GGC TTG ACT GG
BRAF-E14-R	CCT ATA CAT GCA TGC ACA ATC C
BRAF-E15-F	TCA TCC TAA CAC ATT TCA AGC C
BRAF-E15-R	TTT GTG AAT ACT GGG AAC TAT GAA A
BRAF-E2-F	GAG GAA CAC TGG CAG TTA CTG TG
BRAF-E2-R	TCT CTT CCC AAA TCT ATT CCT AAT CC

(*Continued*)

Primers names	Primers sequences
BRAF-E4-F	TGG TGT TGT ATC TGA CCT AGT AAC CC
BRAF-E4-R	CCT CAT ATG GCC TAC AGT ATT TCT TC
CARD11-E10-F	GTA AGA AGC GAG TCG CAG GA
CARD11-E10-R	TTG TAC AAG AAG ACC GGG GC
CARD11-E13-F	TAA GGA GGC TTG GCA TGG TG
CARD11-E13-R	AAT TGA GTC CCT GCG TGT GT
CARD11-E16-F	CTT CTC GGA GCC AGG ACA C
CARD11-E16-R	ATG GCT GAA TGC AAG GCT TAG
CARD11-E19-F	AGA CAC CAA CCC CTT GTC CT
CARD11-E19-R	AGT CCT CGA AGG CAA GCA TC
CARD11-E20-F	AAG AAA GCG TTA GCA TGG AGG
CARD11-E20-R	GAC AGG AGA TGT GAG GCA CTG
CARD11-E23-F	ATT TAA ACA TGG CTG GAA GGG
CARD11-E23-R	AAA CAA CTC TTC ATC CCA CCC
CARD11-E4-F	GAT TTA CGT CAT CTG GGC TCC
CARD11-E4-R	GAA CCA TGC CAC TAC TGA GGA C
CARD11-E5-F	AGT GAG TGA ATG AAT GGC ACC
CARD11-E5-R	TTA TGG GAG AAT TGA GCC CTG
CARD11-E6-F	GAG GCA CGG AGC TTA CAG TT
CARD11-E6-R	AAA CCC ATC AGA GAA GGG CG
CARD11-E8-F	CGA GCA GAG AAC AGC TTT CAG
CARD11-E8-R	AGG AGT GAT CCC TGC ATT GTC
CARD11-E9-F	GAA GGT AGT GAC TGA CAC GCC
CARD11-E9-R	GGA GCC ATT CAT TGC ACA G
CD58-E1-F	CGT AGG CGG TGC TTG AAC
CD58-E1-R	CTG ACT CCA TGA GGG ACA CTG
CD58-E2-F	TTG TGT CAG CAG TTT GTC AGC
CD58-E2-R	TCT CAT AGA CCA TTA ATT TGT GCT AGT C
CD58-E3-F	TGT CTG CTC ATC CTT TCA AGC
CD58-E3-R	GAA AGC CTC CAT GAC ACA TTT A
CD58-E4-F	TCT GAG AAT GAC TGC TTT GGG
CD58-E4-R	AGC AGT CCC ACA CAC GTA AAG
CD58-E5-F	TGA ACA ACT CTT TGG ACC AGC
CD58-E5-R	ATC TGG CTT CCC AAG TAA TGG
CD58-E6-F	ACT CAA GGC AGT TCT TCC TCC
CD58-E6-R	GCA GCT GCT TCA AGT TAC ATT TC
CD79B-E3-F	AAG GAC TAA GCC CAG GGA GTC

(*Continued*)

Primers names	Primers sequences
CD79B-E3-R	ACC ATC ACC ACA AGA GGC AG
CD79B-E4-F	GCA GAA GTG CAA CAA CAC CTC
CD79B-E4-R	ACC AAG AAG GCC ACA ACG AG
CD79B-E5-F	TGT TCT TGC AGA ATG CAC CTC
CD79B-E5-R	TAG GTG GCT GTC TGG TCA ATG
CD79B-E6-F	CACCTACGAGGTAAGGAGAGGG
CD79B-E6-R	CTG GAG ACA AAT GGC AGC TC
CD83-E3-F	AAC ATA AGT TGA GGC TGG ATC TTC
CD83-E3-R	TTT CAG CCA CAG ATG TGA TCC
CIITA-E11-1-F	GTG TCA CAT GTC TGT GGT CCC
CIITA-E11-1-R	AGC ACA GCA ATC ACT CGT GTC
CIITA-E11-2-F	AGA CAC GAG TGA TTG CTG TGC
CIITA-E11-2-R	GCT GTG GCT GTG ACT GAG AAG
CIITA-E11-3-F	AGT GGC GAA ATC AAG GAC AAG
CIITA-E11-3-R	GCA GTT AAT GCC CAA CAC AAG
CIITA-E17-F	TCT GGA ATC TAG GGA TGG TGG
CIITA-E17-R	GCA AGA GAA ACT CAC CTT GGG
CIITA-E18-F	AAT AAC TGC ATC TGC GAC GTG
CIITA-E18-R	AGG GAA GAG CAT GAT TTG AGC
CIITA-E1-F	GCT AGT GAT GAG GCT GTG TGC
CIITA-E1-R	CTC CGG TGG AAG GAA ATA GAC
CIITA-E2-F	ATG TGG CTT GCT CTC TCC AC
CIITA-E2-F	CCA GCT GGG AGT TGT TGT AGG
CIITA-E2-R	GGA CCA GAA TTT CCC CTG AT
CIITA-E2-R	AAT TTC TGG AAA GAG GGC TGG
CIITA-E3-F	TGC TGT AGA GAC GGC AAT CAG
CIITA-E3-R	TTG CAA TGA TTT CTG TGG GAG
CIITA-E4-F	CCT GGA GTG TCA GTG TTC ATT C
CIITA-E4-R	GTC GTC AGG TAT TAG GGC ACC
CIITA-E7-F	AGA CAT CCA TGC CAC TCC AG
CIITA-E7-R	CCC TGC CTT AGT GTC AAC TCC
CIITA-E8-F	GGG CAG GAC AGA AAC AGC TAC
CIITA-E8-R	GAC AGA GAA AGT TGT GCT GGG
EP300-E2-F	CCT CAA TAT GGG AGT TGG TGG
EP300-E2-R	ACA ATG TAA GGC AAA CCC TCC
EP300-E8-F	CCC TGC CTA GCT CCT TAA TGC
EP300-E8-R	ATG ATG GTG GGA AAG GTT GAG

(Continued)

Primers names	Primers sequences
EP300-E25-F	CAG GCA TGA AAG CAA GGT ATC
EP300-E25-R	TTG CAT TTC CAA ACC AAA CAC
EP300-E27-F	GGT ATC TAT ATC AAC TCC AAC TTG TGG
EP300-E27-R	GAA TGG CAT GAA CCC AGG
EP300-E30-F	CCA TGG TGG GAT AAT TGC TTG
EP300-E30-R	AAA TAC GTG GCT GCA TGG C
EP300-E31-F	ATG CCT TCA CAA TTC CGA GAC
EP300-E31-R	AAA GCA TTG AAT TCT GGT CCG
EZH2-E6-F	TTT AGT GCC AGT TAC TAG GCT ATG C
EZH2-E6-R	CAA GCA ATC TGC CCA CCT TAG
EZH2-E8-F	TCG ATG AAT TTG ATT CTT GAT AAC AC
EZH2-E8-R	TTC TAG TTG TAA TAA ATG ATA GCA CTC TCC
EZH2-E9-F	GGA GGA GGA ATG GAG AAT ACG
EZH2-E9-R	CAT AAC AGC ATG GGT GCA GAC
EZH2-E10-F	ATT ATT TGT GAT AAA TGG ATA ATG TGA
EZH2-E10-R	GCA GGA AAC AGA AAC AAC ACA
EZH2-E20-F	CAG CAG GCT TTG TTG TGT TAA G
EZH2-E20-R	TTC AAC AAG GAC AAG TTC AAG TAT TC
FAT4-E1-1-F	CCG CGC CAG GTG TTC CAA GT
FAT4-E1-1-R	CGT CCT GCA GGC GAT AGC GG
FAT4-E1-2-F	CGC CAC CGA CTC GGA CAT CG
FAT4-E1-2-R	CGG TGA GCA GAG CCA CCA CG
FAT4-E1-3-F	GGC GCC TCC GGG AAG CTA TG
FAT4-E1-3-R	GAC TGG GGA GGG GAG CCC AG
FAT4-E1-4-F	TGC TGG GGA CAG GTC TCG GT
FAT4-E1-4-R	TCC AGG GGC CCA AGC AGA CT
FAT4-E1-5-F	AGG TAG TGG CCA GTG GGG GC
FAT4-E1-5-R	TGC CCA CAA ACG ACC CAG CC
FAT4-E1-6-F	GGC GGA GAG GGA CTG CTG TG
FAT4-E1-6-R	TGC ATC AGG GTC ATG TGC AGT CA
FAT4-E3-F	AAA GCC TTC CTT TGT CAA ATA CA
FAT4-E3-R	TCA TTG ATT AAG AGC AGC CGA
FAT4-E8-F	TTT CTT GCA TTT CTA CCT CAG TTT
FAT4-E8-R	CAC AAT TAG TCT AGC AAG AAA GGG
FAT4-E9-1-F	AAT GGA CAA GGA CAG TGG ACC
FAT4-E9-1-R	CAG TCC ACC CAG AAT CAT GTG
FAT4-E9-2-F	TTC CTA TTA TTT AGA TTG CCC TGA AC

(*Continued*)

Primers names	Primers sequences
FAT4-E9-2-R	AAC TCT GAT TAA CTT CGT ACC AAC C
FAT4-E9-3-F	GGG TGG ATT TCA GTA GCA TCC
FAT4-E9-3-R	ACG CAA TCA CAG CAT TTG TTC
FAT4-E9-4-F	TGG AAC AAA TGC TGT GAT TGC
FAT4-E9-4-R	CTT CTT ATT GAT CTG GAA ACC CTT C
FAT4-E9-5-F	ATG GTA ACT TGT TTC CCG GTG
FAT4-E9-5-R	CGT AGA CAG CTC CCT CCA TTC
FAT4-E15-F	TGC ATT TAA ACA TGT GAA ATC AAC T
FAT4-E15-R	CCC AGC ATT AGT CGG AAC TCT
FAT4-E17-1-F	CCT CAG TGA AGA TTG GCT GC
FAT4-E17-1-R	TCC TCA CAG TCA TGT CAT CCC
FAT4-E17-2-F	GGT CCT TAG CCT GAT CCT GTG
FAT4-E17-2-R	GAG ACA GAG GGT TGG GAG TTG
FAT4-E17-3-F	TCC ACT GGA ATC TTC TCC TCC
FAT4-E17-3-R	TTT AGT TGT CCC AGC TTT GCC
FAT4-E17-4-F	CTG AAG CCT CGA AGG TAC CAC
FAT4-E17-4-R	AAA TGC TGC ATG CCA CAT AAC
IRF4-E2-F	GCT TCG CAG CCT CAA AGA CTC
IRF4-E2-R	TCC TCC TCG CGG TTG TAG T
IRF4-E3-F	TGAGCTATCATCGTGCCACTG
IRF4-E3-R	ATA ACT CAG GCA AGG AGG CTG
IRF8-E2-F	TGCAAACCTCTGAGTTTCCTG
IRF8-E2-R	TCA GCA CTC CTG AAC CAA TG
IRF8-E3-F	AAG ATT CAT GGG AAG GGA AGG
IRF8-E3-R	GAC CCA AGT TCT GTG GTG AAG
IRF8-E4-F	AAT TCT GTG CCT TTC CCA AAC
IRF8-E4-R	GGC TGC CTT CAT ACA GCA AG
IRF8-E7-F	TCT TGT CAC AGC ATT CTC CC
IRF8-E7-R	AAC TGG CTG GTG TCG AAG AC
MEF2B-E2-F	AAA GGC TGA CAT GGC ATC AG
MEF2B-E2-R	GAC AGA GGA GAG GTG TGA GGC
MEF2B-E3-F	TAA AGC ACG TCA GCC ACA AAG
MEF2B-E3-R	GCA TGT TCG AGG AAG GGT TAG
MLL2-49415846-F	AGG GGA GGC CAG AGA AGA TA
MLL2-49415846-R	ATG ACG AGG AAG GAG GGA TT
MLL2-49418430-F	CAT CAC AAG CTC ACC GTT TG
MLL2-49418430-R	TGT GGA ATC GCA TCA TTG AG

(Continued)

Primers names	Primers sequences
MLL2-49420076-F	AAC AGT GAC CCT GGG AGA AA
MLL2-49420076-R	GAA GCA AAT CGC TAG CAT CA
MLL2-49420213-F	GCC CGT TGT TCT CAC CAA TA
MLL2-49420213-R	TGC CCC AAT GTC TAC CAT TT
MLL2-49420597-F	ATG GTA GAC ATT GGG GCA AC
MLL2-49420597-R	GAA AGG AGT GCG CTG GAA G
MLL2-49420623-F	AGG GAG CAC TTG GTT AGC AG
MLL2-49420623-R	AAGGTGAAGATTCCCGTCCT
MLL2-49420890-F	CCA CTG CCC TTC TGG ATG
MLL2-49420890-R	TAC AGG GCA CCC TCC TAC A
MLL2-49420960-F	CGC ACT CCT TTC CAT TTC TT
MLL2-49420960-R	TGA GGA GCT GGC ACT AAT GA
MLL2-49421680-F	TCC CAA AGC ACT GGG ATT AC
MLL2-49421680-R	TAT GAG GTG CTG TTC CCA GA
MLL2-49421830-F	CAA ACT GCT TCA GCC AAT CA
MLL2-49421830-R	GGG GAG TGG GTT CTA GGT GT
MLL2-49422918-F	GTA ACA CTT GGC CGC CTC T
MLL2-49422918-R	GGG GTA CAG GTT GCC TCA TA
MLL2-49425021-F	ACC CAG GCT CAC TCA TTC TG
MLL2-49425021-R	GGA GCC CAG TCA GTG AAG A
MLL2-49425220-F	TTC CTG GTG CCC CTA TTG G
MLL2-49425220-R	AGC CCT CAA GAG CCA AAG AG
MLL2-49425625-F	TGG ATG GGT GGG AGG GAG
MLL2-49425625-R	GCA CTT AAG TCC TCA GCA GC
MLL2-49425673-F	CTC TTT GGC TCT TGA GGG CT
MLL2-49425673-R	TGG GGT TGG AAT CAT GCC TA
MLL2-49425853-F	CAA GTT GAG GTT GGC AGC C
MLL2-49425853-R	ATC AGC CTG GGT CCA TGA C
MLL2-49426159-F	TGT GGC CCT GTA TTA TTT TGC A
MLL2-49426159-R	CTC TAC ACA TCA GGG AGG GC
MLL2-49426471-F	TTT TGT TCC TTG CCC GTC AG
MLL2-49426471-R	AACAACAGCACCAACAGCAA
MLL2-49427057-F	CTT GCT GTT GGT GCT GTT GT
MLL2-49427057-R	TCT AGG ACA GGT GGC AAT CC
MLL2-49427364-F	CTG TTG CTG CTG GAT TGC C
MLL2-49427364-R	CTC ACC AAG CTC CCT GGT C
MLL2-49428035-F	TCT TGC TCT GCT GCT CTG TA

(*Continued*)

Primers names	Primers sequences
MLL2-49428035-R	GAG TGC TGG TGA TCC CTC C
MLL2-49428427-F	GAG GGA TCA CCA GCA CTC C
MLL2-49428427-R	AGG TAA GAC TCA GGG CTG GA
MLL2-49430941-F	AGC CAA AGT TCT TTG TGT CCC
MLL2-49430941-R	CCA CTG ATG CCC ACC CAG
MLL2-49431115-F	CAT TGC CAA TTG CTG CG
MLL2-49431115-R	TGT CTT TGC CAC ATG AGG G
MLL2-49431358-F	CCT GGC TGT CGG GCA CCT
MLL2-49431358-R	CCT GGT GGA CCT GCA GCA TC
MLL2-49431709-F	TCA TGC TCT GTC CTG GCT TT
MLL2-49431709-R	GAG AAG GCT GAA CGG GAG G
MLL2-49431742-F	AGG CCT TCT CAG CTG TGT G
MLL2-49431742-R	CTT CAA TGA GCA CCT GAG GC
MLL2-49431925-F	AAT TGG CAA GGA GAA GGG TG
MLL2-49431925-R	TGA AGA GCT TGC TCA CCT GG
MLL2-49432721-F	ACG CCT TCC TCT ATG GAT GTG
MLL2-49432721-R	TTC TGT ACA TTG TGC CGC AG
MLL2-49433749-F	GTTT CAC TTT CCC TCA GGC AG
MLL2-49433749-R	ATC AGC AGC TCT CGT AGT CGC
MLL2-49434438-F	GAG CTG GAC TGG GAC TGA G
MLL2-49434438-R	CTG GGC TTT GTT GAC TCA CC
MLL2-49434910-F	AGC TCT TCC TTC TTC ACC TCT
MLL2-49434910-R	TCT TCC TCA AGC TCC CAC C
MLL2-49435230-F	TAA GGC TGT GTC CCA TAT CCC
MLL2-49435230-R	GGA GCT TGA GGA AGA GCT CAC
MLL2-49436557-F	GGA GGA GGT CCT ATC TTT GGG
MLL2-49436557-R	CCT ACA AGA CGG ACA GGA TCA G
MLL2-49438195-F	TCT CAG TGG CAG GAT GAC AAG
MLL2-49438195-R	ACC ACC AAT GCC TAT GAG GAG
MLL2-49438548-F	TGT CAT CCT GCC ACT GAG AG
MLL2-49438548-R	ATG CAG AGC CCC AGT ACT TT
MLL2-49439859-F	ACC TGG CTC TTT CAC CTT CA
MLL2-49439859-R	TGA ACG GTG AGA ACA TCC CT
MLL2-49440431-F	GGT TGA AAC TTG CAG TTC TGG
MLL2-49440431-R	AAC TGA GTG GCA ATG TAG CCC
MLL2-49440432-F	CAC TTG TAC CTG AGA GGC AGA G
MLL2-49440432-R	CCC AGC CTC TGT CAC ATA CTC

(*Continued*)

Primers names	Primers sequences
MLL2-49443789-F	ATC CGT AGA GAC CCC CAA CT
MLL2-49443789-R	CTC TGG ATG GGA TTG ATG CT
MLL2-49444297-F	GTG CAG CTC AGC CTC ATC T
MLL2-49444297-R	TCT CAC CCA TCA TCA CAG CT
MLL2-49444408-F	CTG GGA AGG AGG GGA GTT TT
MLL2-49444408-R	CAG CTG TAA CTC TTC ATG GCA
MLL2-49444855-F	GGA CAA CGG CAG CTC CTC
MLL2-49444855-R	CTG AGG GAC CAC ATC TGT CC
MLL2-49445257-F	GCA TCA CGC CTG TCC CCA CC
MLL2-49445257-R	GTG GCT CCT CAG GCA CAG CG
MLL2-49445610-F	CAC TTG AGA CGC CTC TAT CCC
MLL2-49445610-R	CTC AGG TAG TGG CAA CAG GG
MLL2-49446078-F	GGA GAC AAG GGC GAC TCC
MLL2-49446078-R	GGC AAG AGA CAG TTT GTG CA
MLL2-49446703-F	CAA ATC CTA GAG AGC ACA CTG G
MLL2-49446703-R	TTT GCT GTC TGA CCA ATG CC
MLL2-49447867-F	AAA ATC CAA GGC ACA TTT GG
MLL2-49447867-R	GGC TGG TGG GCT TCT GAG
MYOM2-E2-F	TCT GCA TAT ACC AGT GGT TTG G
MYOM2-E2-R	AGC TAA GGT CGC TCT TCC CTC
MYD88-E3-F	GTA TCA TCT TGG GAA GGG TGC
MYD88-E3-R	AGC TAG GAG GAG ATG CCC AG
MYD88-E4-F	CTG GCA CCT GTG TCT GGT CTA
MYD88-E4-R	TTG GTA CTG CAT CCA CAG TCC
MYD88-E5-F	GAT GGC TGT TGT TAA CCC TGG
MYD88-E5-R	TCC TAC AACGAAAGGAGGAGG
MYOM2-E14-F	GCG AGA ACA GAG CAT TTC TTC
MYOM2-E14-R	CAC ACA ATT TAA GGC TAT TTG GTG
MYOM2-E17-F	GTT GCT TCC TCC TCC CTC TG
MYOM2-E17-R	CAT CAA ACG CAG AAA CAT TGC
MYOM2-E19-F	AAC AGA TAC GGA TGG TCC TGG
MYOM2-E19-R	GTG ATT GGC CCG CCT TTA C
MYOM2-E21-F	GTC CCT AAG CCG CTC ACT TC
MYOM2-E21-R	AAA CAG GCA AGG TTC TGC CA
MYOM2-E28-F	TTG ACG TTA TTG GGA GCG AC
MYOM2-E28-R	ATT CAC CGT GGT CCT TCT TTG
MYOM2-E31-F	GTG AGA ACA GGA TTG CAG GTG

(*Continued*)

Primers names	Primers sequences
MYOM2-E31-R	GAA ATG GCA GTG CCT TGA TG
MYOM2-E32-F	GTC ACA GCA ACG GGA CAT TG
MYOM2-E32-R	TGC TGG AAT TCT GCA AAT GCT T
PDL1-E2-F	CCG GTG TTA CAT CTG CAA GG
PDL1-E2-R	CAG AGC AGG CTG AAT TGT TTC
PDL2-E2-F	CTC ATA GAT GGC CAG AAA CGG
PDL2-E2-R	GGG AAG AGG AGG ATC TGG AAG
PIM1-E1-1-F	CCC GAG GGA GTC GGT GGC A
PIM1-E1-1-R	CTT GGT GGC GTG CAG GTC GT
PIM1-E1-2-F	GCA TAG CCT TCG GCA CAG
PIM1-E1-2-R	CCT AGG AAA GGG GGA AGC AC
PIM1-E2-F	CAC TGA GTC CCC GTG CTT
PIM1-E2-R	TCA CCC CAC CCA CTCATC
PIM1-E3+4-F	GTG CTT TAG CCC GGA CGA GG
PIM1-E3+4-R	GAC GGT GTC CTT GAG CAG CG
PIM1-E4-F	CAG CTT CTT CTG GCA GGT G
PIM1-E4-R	AAA TCC CCG GCT TTA CTA CG
PIM1-E5-F	ACC CAG CTT CTT TGT GCT TG
PIM1-E5-R	GGA GAG TGA AGC TCT CCA GAA
PKD1-E11-F	CTC TCC ACA GGT CAC CCT CC
PKD1-E11-R	AAC AGG GTA TCG TTG GTC TCC
PKD1-E15-1-F	CTG CCA ACC ACA CCT ATG C
PKD1-E15-1-R	ACC TCC AGG ACG AAG ACC AG
PKD1-E15-2-F	CTA CAC CTG GGA CTT TGG CAC
PKD1-E15-2-R	AGC GGA AGG TGT AAG AGA TGG
PKD1-E15-3-F	ACC ATC TCT TAC ACC TTC CGC
PKD1-E15-3-R	GCT ATG GGT GGT AAA TGG CTC
PKD1-E15-4-F	GAG CCA TTT ACC ACC CAT AGC
PKD1-E15-4-R	GTG AGG TTG TAC GTG GCT GAG
PKD1-E15-5-F	CTG AGA GGA ACT TCA CAG CCC
PKD1-E15-5-R	CAG TCT GGT AGG TGA CGC AG
PKD1-E16-F	ATG AGC CCA GAG AAC ACC C
PKD1-E16-R	GAC TGT ACG TGG AAC TGT GGC
PKD1-E23-F	GGA GCC GAG TCA CCA TCT C
PKD1-E23-R	TCC AGC AGC GTA TAG TTG AGC
PKD1-E25-F	GTA TGG GCT CTG AGA CTG CG
PKD1-E25-R	AGG ATA GAG CCG AGC CCA C

(Continued)

Primers names	Primers sequences
PKD1-E45-F	CTT GGG CCT GGT GGT GCT CG
PKD1-E45-R	ACT GGG CCG TAC CCA CCT CC
PKD1-E46-F	CAG GCT GCG CCT CTG GAT GG
PKD1-E46-R	GCC AGT GGC CAG GTC CAC AC
PRDM1-E2-F	TCA GAA GGA GCC ACA GGA AC
PRDM1-E2-R	AAG GCC TCA AGG GCA AGT AG
PRDM1-E3-F	TTT AAT GCT TGG TTG AGT ATT TGC
PRDM1-E3-R	AAA GGT GAG CCA CCC AGC
PRDM1-E5-1-F	GTG TCG CCA GCT GTT ACT CAG
PRDM1-E5-1-R	CGT AGG ACG CGT TCA AGT AAG
PRDM1-E5-2-F	CCC AAA GAG AGA GTA CAG CGT G
PRDM1-E5-2-R	AGT TGT TGA TGC CAT TCA TGC
PRDM1-E5-3-F	TAC TTG AAC GCG TCC TAC GG
PRDM1-E5-3-R	ATC TTG CCG TTC TGC TTC TTC
PRDM1-E7-F	CAC AAG GAG GCT TCT CAC CTC
PRDM1-E7-R	TTT GCA AAG ACA CTT TCA GGC
TNFAIP3-E2-F	CTG CAG GCA GCT ATA GAG GAG
TNFAIP3-E2-R	GAT TTG AGT TTG GGC TTG TCC
TNFAIP3-E3-F	CTC AGT ACA TGT GGG GCG TT
TNFAIP3-E3-R	GGG GGA AAA ACC TAC CCG AG
TNFAIP3-E4-F	CTC GGG TAG GTT TTT CCC CC
TNFAIP3-E4-R	GAG CCA AGG CAT AAG GCT GA
TNFAIP3-E6-F	TGG AGC AAG TAA ACG CCT GT
TNFAIP3-E6-R	AAA CTG GCC GCA AAT CAC TC
TNFAIP3-E7-1-F	TGT GTC AGA TCA TGT TGC GTG
TNFAIP3-E7-1-R	CAA GGG CTC ATA GGC TTC TCC
TNFAIP3-E7-2-F	GTT CAG TGA GAC CAC TGCCA
TNFAIP3-E7-2-R	GCT CTG TCT ACA GGG CAC TC
TNFAIP3-E9-F	TGA GAT TTC ATT GTG CTC TCC C
TNFAIP3-E9-R	CTCAGA CAC CCT TAA GCC CAC
TNFSF9-E3-R	GTA GAG AAA GCA GCA GGG ACC
TNFSF9-E4-F	CTA AGT GCA TGC TTT CCT CCC
TP53-1-F	TAA GCAGCA GGA GAA AGC CC
TP53-1-R	TCC TAC AGT ACT CCC CTG CC
TP53-2-F	AGG AGA GAT GCT GAG GGT GT
TP53-2-R	GAG ACC TGT GGG AAG CGA AA
TP53-3-F	GCC ACA GGT CTC CCC AAG GC

(*Continued*)

Primers names	Primers sequences
TP53-3-R	TGG GGC ACA GCA GGC CAG TG
TP53-4-F	TTG GGA GTA GAT GGA GCC T
TP53-4-R	AGT GTT AGA CTG GAA ACT TT

Fluorescence *in situ* hybridization (FISH) analysis

MYC translocation and amplification were investigated by FISH analysis on FFPE sections of 196 cases using the Vysis LSI MYC Dual Color Break Apart Rearrangement probe and Vysis IGH/MYC/CEP 8 Tri-Color DF FISH Probe Kit (Vysis), as described previously [1]. Amplification of *MYC* was defined by the presence of more than two orange signals of *MYC* along with two aqua signals of centromere 8. Samples were analyzed on a fluorescence microscope and images were captured with the Isis software (META systems). At least 200 intact, non-overlapping nuclei were scored for each case, and the normal cut-off value was 10% of suspected tumor cells.

Gene copy number variation analysis based on NGS data

A total of 203 samples have been included for copy number variation (CNV) analysis based on NGS data, which included 196 DLBCL samples and 6 reactive lymph nodes. One DLBCL sample was used in duplicate for the purpose of quality control of the analysis. Candidate genes with genomic gain or loss were detected based on the read-depth information of the NGS data [2-5]. First, we aligned Ion amplicon sequencing reads to hg19 using the BWA-MEM algorithm and calculated the average depth for each target region across 203 samples [6]. Second, the target regions were removed due to abnormal read-depth if larger than the 99% quantile or smaller than the 1% quantile, read-depth below 20 × in either of DLBCL samples and reactive lymph nodes or read-depth ratio greater than 4. The read-depth (d_i^j) of gene *j* of sample *i* as well as the read-depth of sample *i* (D_i) were then calculated based on the mean depth of those retained target regions. Third, the read-depth ratio (R_i^j) of gene *j* of sample *i* was calculated according to one of the formulas listed below. If the distribution pattern of d_i^j was significantly different from that of D_i in 203 samples, d_i^j was then deemed to indicate over- or under-sampling in the Ion amplicon sequencing experiment. Accordingly, the read-depth ratio (R_i^j) would be calculated with the correction coefficient *r* as follows:

$$R_i^j = \frac{d_i^j}{D_i} \times r, r = \frac{\overline{D}}{\overline{d^j}} \quad (1)$$

where d_i^j is the average read-depth of gene *j* in sample *i*, D_i is the average read-depth of sample *i*, $\overline{D}$ is the mean value of D_i, $\overline{d^j}$ is the mean value of d_i^j in 203 samples and *r* is the correction factor. If the distribution pattern of d_i^j was not significantly different with that of D_i in 203 samples, the read-depth ratio (R_i^j) would be calculated without correction, as follows:

$$R_i^j = \frac{d_i^j}{D_i} \quad (2)$$

Finally, we generated the normal distribution of the read-depth ratio for individual gene with their mean and standard deviation based on R_i^j values ranging from 0.8 to 1.2. To detect the potential gene copy number loss or gain in an individual sample, a *P* value was calculated by comparing R_i^j with the generated normal distribution of the read-depth ratio. The *Q*-value was corrected from the *P*-value with FDR. Loss of gene copy number in a sample was defined as the mean depth ratio of a sample lower than the normalized mean depth ratio with *Q*-value < = 1e^{-2}. If the mean depth ratio of a sample was higher than the normalized mean depth ratio with *Q*-value < = 1e^{-5}, a gain of copy number of a given gene was considered. All identified loss of gene copy number had been ruled out when the sample contained detectable heterozygous single nucleotide variations with mutant allele frequency between 0.4 and 0.6. CNV frequencies of all 27 genes were further verified in the COSMIC cancer database (http://cancer.sanger.ac.uk/cosmic).

Immunohistochemistry

Paraffin embedded tissues sections were dewaxed in xylene and subjected to immunohistochemical staining analysis as described elsewhere. Anti-p105/p50 (NF-kB1), anti-p100/p52 (NF-κB2), anti-phospho-p38 MAPK (Thr180/Tyr182) rabbit monoclonal antibody (Cell signaling Technology), anti-TP53 rabbit polyclonal antibody (Proteintech), anti-CD58/LFA-3 goat polyclonal

antibody (R&D Systems) and biotinylated secondary antibody were used in the present study. Thermo Scientific™ UltraVision Quanto Detection System HRP DAB and Polink-1 HRP Goat DAB Detection System were used to detect immune reactivity. In order to define elevated TP53 protein expression, a cut-off was set at 50% positive staining cells as proposed by Xu-Monette et al [11]. For NF-κB and P38-MAPK activity, cases were considered to be positive when ≥ 30% of tumor cells showed nuclear NF-κB localization. Each slide was evaluated independently by two pathologists who were blinded to clinical and outcome data.

REFERENCES

1. Hans CP, Weisenburger DD, Greiner TC, Gascoyne RD, Delabie J, Ott G, Müller-Hermelink HK, Campo E, Braziel RM, Jaffe ES, Pan Z, Farinha P, Smith LM, *et al*. Confirmation of the molecular classification of diffuse large B-cell lymphoma by immunohistochemistry using a tissue microarray. Blood. 2004;103: 275-282.
2. Xie C, Tammi MT. CNV-seq, a new method to detect copy number variation using high-throughput sequencing. BMC Bioinformatics. 2009;10:80.
3. Plagnol V, Curtis J, Epstein M, Mok KY, Stebbings E, Grigoriadou S, Wood NW, Hambleton S, Burns SO, Thrasher AJ, Kumararatne D, Doffinger R, Nejentsev S. A robust model for read count data in exome sequencing experiments and implications for copy number variant calling. Bioinformatics. 2012; 28: 2747-2754.
4. Koboldt DC, Zhang Q, Larson DE, Shen D, McLellan MD, Lin L, Miller CA, Mardis ER, Ding L, Wilson RK. VarScan 2: somatic mutation and copy number alteration discovery in cancer by exome sequencing. Genome Res. 2012; 22:568-576.
5. Sathirapongsasuti JF1, Lee H, Horst BA, Brunner G, Cochran AJ, Binder S, et al. Exome sequencing-based copy-number variation and loss of heterozygosity detection: ExomeCNV. Bioinformatics. 2011; 27: 2648-54.
6. Li H. Aligning sequence reads, clone sequences and assembly contigs with BWA-MEM. arXiv preprint arXiv:1303.3997, 2013.

SUPPLEMENTARY FIGURES AND TABLE

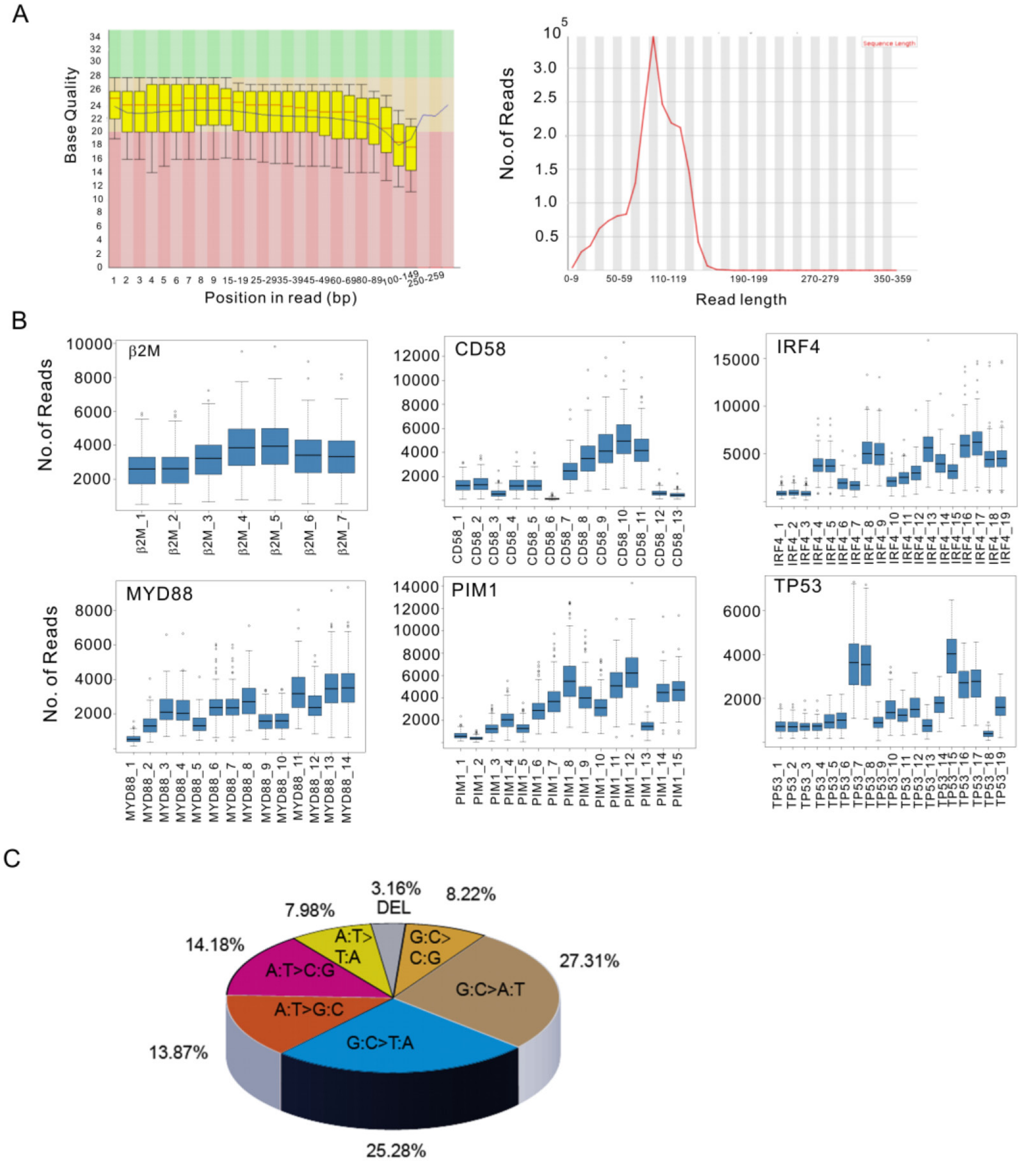

Supplementary Figure S1: Next generation sequencing data output. A. Distribution of generated amplicon sequence reads per patient. For an exemplary patient, the read length (x-axis) and number of generated sequence reads (y-axis) is represented. **B.** Coverage of generated sequence reads and distribution of generated sequence reads per amplicon. For each of the 27 amplicons (x-axis), the range of generated sequence reads is represented by box-and-whisker plots (y-axis). **C.** Proportion of base alterations in DLBCL genome. Diagram depicts the proportion of DLBCL somatic mutations in each mutational class of transitions and transversions.

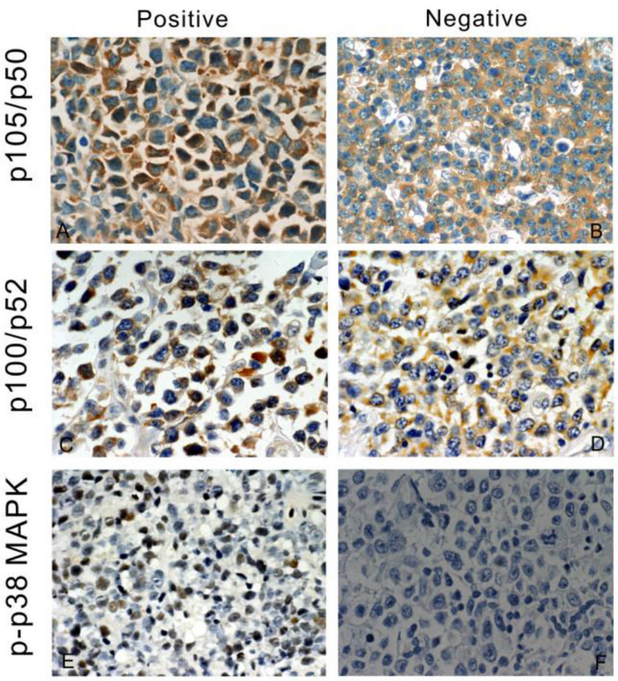

Supplementary Figure S2: The NF-κB pathway and p-38 MAPK pathway are activated in primary DLBCL of the CNS or testis with *MYD88* mutations. Immunohistochemical staining of primary DLBCL of the CNS or testis biopsy with anti-NF-kB1 (p105/p50, A-B), anti- NF-kB2 (p100/p52, C-D) and anti- p-38 MAPK (E-F) antibodies. Nuclear location of NF-kB denotes active signaling, as opposed to the inactive, cytoplasmic complex. Original magnification, × 200.

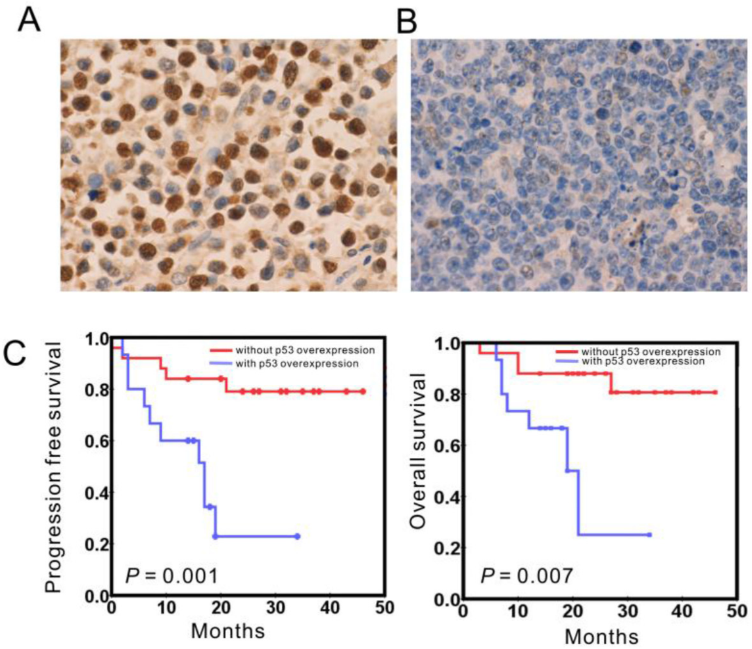

Supplementary Figure S3: p53 overexpression was associated with mutational status and predicted worse PFS and OS. A total 40 DLBCL cases were included to detect p53 expression using immunohistochemical staining, of which, 17 cases with *TP53* mutations and 23 cases without. **A.** Elevated p53 expression was observed in a reprehensive case with *TP53* mutation, original magnification, × 200. **B.** A reprehensive case without *TP53* mutation showed no elevated immunoreactivity for p53, original magnification, × 200. **C.** p53 overexpression predicted shorter PFS and OS.

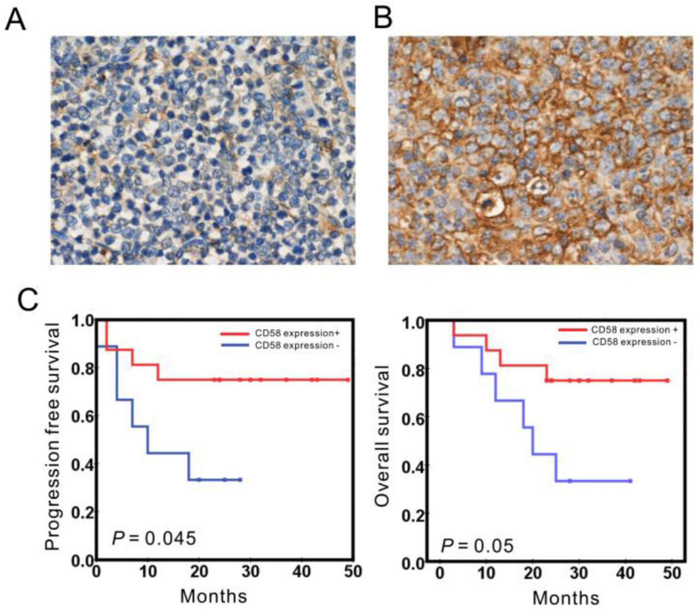

Supplementary Figure S4: *CD58* mutated DLBCL cases lack CD58 cell surface expression and have inferior PFS and OS. CD58 protein expression was detected in 10 cases with *CD58* mutation and 15 wild-type cases. **A.** A reprehensive *CD58* mutated case showed defective CD58 surface expression, original magnification, × 200. **B.** CD58 surface expression in a reprehensive CD58 WT case, original magnification, × 200. **C.** *CD58* mutated DLBCL cases had inferior PFS and OS.

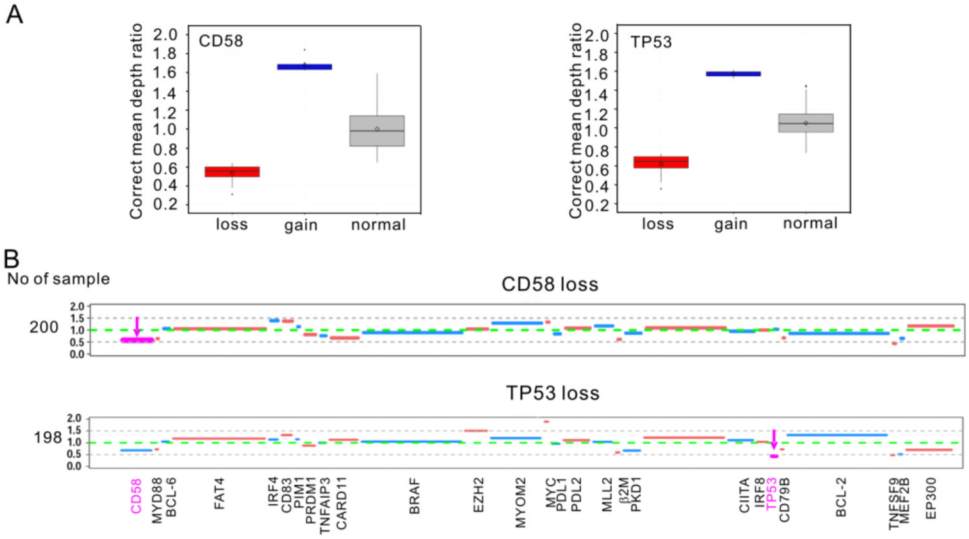

Supplementary Figure S5: Detection of gene copy number losses in DLBCL samples based on next generation sequencing data. A total 203 samples have been included for copy number variation (CNV) analysis based on NGS data, of which included 196 DLBCL samples and 6 reactive lymph nodes. One DLBCL sample was set as duplicate for the purpose of quality control for the analysis. Candidate genes with genomic gain or loss were detected based on read-depth information of NGS data. **A.** The distribution of depth ratio in different sample groups with loss, gain or normal gene copy number. The mean depth ratio of 203 samples (horizontal line) is set as 1.0 with standard deviation ranging from 0.8 to 1.2. The Loss of gene copy number in a sample is defined when the mean depth ratio of a sample is lower than the normalized mean depth ratio with *Q*-value ≤ 1e-2. If the mean depth ratio of a sample is higher than the normalized mean depth ratio with *Q*-value ≤ 1e-5 was considered as gain of a given gene. Colored box represents the mean depth ratio of samples with loss (red), gain (blue) or normal gene copy number (grey) with standard deviation. **B.** Representative results showing losses of *CD58* or *TP53* copy number detected based on the mean depth ratio (purple lines and arrows). The corrected mean depth ratio calculated from whole samples (green dotted line) is set as 1.0 with standard deviation ranging from 0.8 to 1.2. The depth ratio of the sample analyzed is shown on the left.

Supplementary Table S1: Frequencies of 27 gene mutations in patients with DLBCL

Genes	Mutated cases	Frequency (%)
PIM1	53	27.04
MLL2	38	18.59
FAT4	34	15.90
TP53	29	14.80
CD79B	29	14.80
MYD88	27	13.78
PKD1	19	9.69
CARD11	17	8.64
β2M	16	8.16
TNFAIP3	14	7.14
CIITA	14	7.14
IRF4	12	6.12
MYC	11	5.61
BCL-6	11	5.61
EP300	10	5.10
CD58	10	5.10
CD83	9	4.59
BRAF	9	4.59
PRDM1	8	4.08
IRF8	8	4.08
BCL-2	8	4.08
MYOM2	7	3.57
MEF2B	6	3.06
EZH2	6	3.06
PDL2	2	1.02
PDL1	2	1.02
TNFSF9	1	0.05
